# Supplementary material for: The role of repetitive thinking and spirituality in the development of posttraumatic growth and symptoms of posttraumatic stress disorder
Source: PLoS One. 2022 Aug 4;17(8):e0272036. doi: 10.1371/journal.pone.0272036 (PMC9352075; doi:10.1371/journal.pone.0272036)
Supplement: Attachment — Submitted filename: Review Article- Repetitive Thinking, Spirituality, PTSD and PTG (Benau, 2021. PDF).pdf [file pone.0272036.s001.pdf]

Review (Ken Benau, PhD)

PLOS ONE

YOU MAY POST MY FEEDBACK, BUT I PREFER YOU NOT USE MY NAME.

OVERALL*: *I RECOMMEND THIS PAPER BE ACCEPTED WITH REVISIONS. SEE BELOW. I BELIEVE THIS RESEARCH IS RELEVANT, BUT THE AUTHORS WOULD BENEFIT FROM A MORE COMPLEX UNDERSTANDING OF THE PSYCHOLOGICAL AND RELATIONAL/ATTACHMENT MECHANISMS THAT CONTRIBUTE TO PTSD AND PTG.

The role of repetitive thinking and spirituality in the development of posttraumatic growth and symptoms of posttraumatic stress disorder

Abstract, and general comment: Why call it "deliberate rumination" rather than "deep, continuous thinking", "reflective thinking", or the like. Rumination has a negative connotation, at least for many psychotherapists. It tends to be associated with anxious thinking that is stuck, "rat on the wheel" thinking, that doesn't get traction and make forward movement. The rumination that comes with PTSD is not the same as "deep, continuous thinking or problem solving".

p. 2-3: I think the definition of trauma should include not only physical/psychological threat, but mind/body *overwhelm*, that is more than the person can bear given their consttitution, developmental status, intra-personal and inter-personal resources, etc. at the time of the event. If no overwhelm, no helplessness and powerlessness in the face of the event, then no trauma.

p. 4: While trauma's impact on cognition and beliefs is very important, it is equally important to recognize the contribution of somatic experience and emotions on the survivor's post-traumatic experience. I would at minimum reference the somatic and emotional domains of experience, in addition to the cognitive/beliefts.

p. 4: Again, intrusive thoughts are always accompanied by intrusive somatic experience and affect. It would important not to leave out the somatic/affective in this discussion, even though it is not the authors' research focus.

p. 4-5: While I don't like the term "deliberative rumination", I think the key to its distinction from "intrusive rumination" is a) DR's *ability to change* rather than remain repetitive and stuck; and 2) the DR person's capacity for *self-reflection*. Without the capacity for self-reflection, that is to think about and give new meaning to traumatic experience, there can be no successful processing of the trauma, and therefore no post-traumatic group (ptg).

p. 5: While perhaps not absolutely necessary, I think it would be interesting if the authors' briefly hypothesize as to why spirituality is associated with lower levels of distress, particularly post-trauma.

p. 6: "1.Controlling for demographic variables, PTSD and IR, it is predicted that ***TST***, DR and spirituality will all show unique positive associations with PTG.

What is "TST"? (Also, on p. 8, p. 14, 21, etc.)

p. 7: "I*nclusion criteria*: University students were eligible to take part in the survey if they were over 18 years and had experienced a traumatic life event after the age of 16 years. This age limit was chosen in an attempt to capture incidents of Type I trauma (rather than complex Type II trauma), and to ensure that participants had cognitively processed the trauma as adults and not as children."

I'd recommend the authors explain why they were focusing on Type 1 trauma. Also, please specify what percentage were Type I vs Type II trauma. I would also consider comparing interpersonal vs. non-human (e.g. car accident, natural disaster, illness) trauma, as that could potentially differentiate with respect to PTG and the effects of spirituality.

p. 9: "The Event Related Rumination Inventory (ERRI; [26]) is a 20-item scale used to assess levels of rumination during the weeks immediately following a highly stressful event."

Since this measure assesses the degree of rumination weeks after the stressful event, how do you know this is an accurate measure 6 months later, for example. The authors should explain how they understand the ERRI is still appropriate 6 months later.

pp. 10-11: With respect to the spirituality measure, the authors should explain if they used the overall score, as contrasted with the subscales, and why, as pertains to their research questions.

p. 17: "Additionally, a significant positive relationship was found between DR and spirituality. This suggests that greater the spirituality, the more likely they are to engage in DR, and conversely, the more someone engages in DR, the more likely they are to report having spiritual beliefs."

It is important the authors' hypothesize why they believe there is a significant positive relationship between DR and spirituality. What is the cognitive, emotional, and/or relational, etc. explanation for the correlation.

p. 17: "This finding suggests that spirituality is a moderator in the relationship between DR and PTG."

It is important the authors' hypothesize why they believe there is a significant positive relationship between DR and spirituality. What is the cognitive, emotional, and/or relational, etc. explanation for the correlation.

p. 20: "However, the moderating relationship reported here supports the theory that having a belief system can provide individuals with a framework that facilitates DR and attempts at understanding and meaning-making in the aftermath of distressing life events [31]."

While this may be true, the authors' should think about other ways DR and spirituality contribute to PTG. For example, might these positive correlations have something to do with a self-reflective capacity, seen in both DR and spirituality? Might spirituality allow a person to feel a connection with God/higher power, and this provides a secure attachment to helps the trauma survivor feel less alone, and therefore less likely to feel overwhelmed by trauma reminders. In short, I'd recommend the authors' consider various psychological variables other than "meaning making" alone that might best explain these correlations. At minimum, I'd recommend the authors' offer alternative explanations/hypotheses explaining the positive correlations, under the rubric of "Further Study" (pp. 21-22).

p. 20: "The subscale of 'existential well-being' appears to be an anomaly amongst the spirituality subsections."

I'd recommend the authors look at the specific items that make up the "existential well-being" subscale. That might give them greater or less support for their hypothesis. For example, I could imagine "existential" might have more to do with the individual alone, than connected with a higher power. If that were the case, then "alone" vs. "securely attached with an higher power" could significantly impact PTSD and PTG.

p. 20: "Consistent with existing literature, a strong positive relationship was found between DR and PTG." There may be other explanations to explain this correlation than the one offered by the authors. For example, DR is associated with a capacity for self-reflection, and this capacity to "think about" the self and the traumatic experience could attenuate the PTSD symptoms and enhance PTG. At minimum, I'd like the authors to hypothesize about possible alternative explanations for the DR/PTG correlation, and include this again under "Further Study" (pp. 21-22).

p. 21: "However, measures of reflective and brooding rumination mostly relate to depression rather than in relation to traumatic events."

I'd recommend the authors reconsider the power of reflection vs brooding as relates to trauma. Reflective vs perseverative thinking is, in my clinical experience, differentially correlated with PTSD and PTG. In short, I would predict the greater reflective capacity, the less PTSD and greater PTG symptoms.

p. 22: "Even if clients do not hold spiritual beliefs, therapy could be tailored to encourage particular thinking styles that are associated with spirituality, such as acceptance [56] to aid reflective rumination and foster psychological growth."

While thinking styles does impact PTG vs PTSD, in my clinical experience and that of many other, emotional and somatic experience, that is subcortical experience, are more important. The authors should at minimum mention this and recommend it for future research.
