# Supplementary material for: The role of repetitive thinking and spirituality in the development of posttraumatic growth and symptoms of posttraumatic stress disorder
Source: PLoS One. 2022 Aug 4;17(8):e0272036. doi: 10.1371/journal.pone.0272036 (PMC9352075; doi:10.1371/journal.pone.0272036)
Supplement: Attachment — Submitted filename: Response to reviewers.docx [file pone.0272036.s002.docx]

Dear Editor

Many thanks for the opportunity to respond to the reviewer comments related to our article “The role of repetitive thinking and spirituality in the development of posttraumatic growth and symptoms of posttraumatic stress disorder”. Please see below each editor/reviewer comment numbered, with our response in bold underneath. Any amendments made are noted in our response here with indicative line numbers, as well as tracked in the manuscript. Any additional amendments made not raised by the reviewers are also noted as well references cited in our response but not included in the manuscript revisions.

We look forward to hearing from you.

Best wishes

Dr Catrin Eames, on behalf of the authors.

Editor comments

1. Please ensure that your manuscript meets PLOS ONE's style requirements, including those for file naming.

**RESPONSE: We have amended the manuscript formatting and file names to adhere to the style requirements.**

2. Please review your reference list to ensure that it is complete and correct. If you have cited papers that have been retracted, please include the rationale for doing so in the manuscript text, or remove these references and replace them with relevant current references. Any changes to the reference list should be mentioned in the rebuttal letter that accompanies your revised manuscript. If you need to cite a retracted article, indicate the article’s retracted status in the References list and also include a citation and full reference for the retraction notice.

**RESPONSE: We have checked all references and have reformatted to meet PLOS ONE’s style requirements. Additionally, we have included some further references in some sections that have been added in light of reviewer comments. These are highlighted in the manuscript as track changes. The reference list as a whole is therefore track-changed.**

3. We note that you have indicated that data from this study are available upon request. PLOS only allows data to be available upon request if there are legal or ethical restrictions on sharing data publicly. For more information on unacceptable data access restrictions, please see <https://hes32-ctp.trendmicro.com:443/wis/clicktime/v1/query?url=http%3a%2f%2fjournals.plos.org%2fplosone%2fs%2fdata%2davailability%23loc%2dunacceptable%2ddata%2daccess%2drestrictions&umid=3ce35460-db02-4393-9388-706b34028785&auth=768f192bba830b801fed4f40fb360f4d1374fa7c-51078a7fdaf219df28bbd0c56eafb651de7a4837>.

 In your revised cover letter, please address the following prompts:

 a) If there are ethical or legal restrictions on sharing a de-identified data set, please explain them in detail (e.g., data contain potentially sensitive information, data are owned by a third-party organization, etc.) and who has imposed them (e.g., an ethics committee). Please also provide contact information for a data access committee, ethics committee, or other institutional body to which data requests may be sent.
b) If there are no restrictions, please upload the minimal anonymized data set necessary to replicate your study findings as either Supporting Information files or to a stable, public repository and provide us with the relevant URLs, DOIs, or accession numbers.

**RESPONSE: When submitting the ethics approval for the study, the ethics committee approved access to anonymised data only to those researchers listed in the ethics application and therefore participants did not provide consent for their data to be used in a shared repository. Upon completion of the study, data stored for 5 years then archived. The anonymised dataset is now archived. Contacts for the archived dataset are:** [**recman@liverpool.ac.uk**](mailto:recman@liverpool.ac.uk) **and ethics committee are:** [**ethics@liverpool.ac.uk**](mailto:ethics@liverpool.ac.uk)**.**

We will update your Data Availability statement on your behalf to reflect the information you provide.


[Note: HTML markup is below. Please do not edit.]

Reviewers' comments:

Reviewer's Responses to Questions

Comments to the Author

1. Is the manuscript technically sound, and do the data support the conclusions? The manuscript must describe a technically sound piece of scientific research with data that supports the conclusions. Experiments must have been conducted rigorously, with appropriate controls, replication, and sample sizes. The conclusions must be drawn appropriately based on the data presented.

Reviewer #1: Partly
________________________________

2. Has the statistical analysis been performed appropriately and rigorously?

Reviewer #1: I Don't Know
________________________________

3. Have the authors made all data underlying the findings in their manuscript fully available?

The PLOS Data policy<<https://hes32-ctp.trendmicro.com:443/wis/clicktime/v1/query?url=http%3a%2f%2fwww.plosone.org%2fstatic%2fpolicies.action%23sharing&umid=3ce35460-db02-4393-9388-706b34028785&auth=768f192bba830b801fed4f40fb360f4d1374fa7c-c980035071b360584114d838c3cb5981f33f266b>> requires authors to make all data underlying the findings described in their manuscript fully available without restriction, with rare exception (please refer to the Data Availability Statement in the manuscript PDF file). The data should be provided as part of the manuscript or its supporting information, or deposited to a public repository. For example, in addition to summary statistics, the data points behind means, medians and variance measures should be available. If there are restrictions on publicly sharing data—e.g. participant privacy or use of data from a third party—those must be specified.

Reviewer #1: Yes
________________________________

4. Is the manuscript presented in an intelligible fashion and written in standard English? PLOS ONE does not copyedit accepted manuscripts, so the language in submitted articles must be clear, correct, and unambiguous. Any typographical or grammatical errors should be corrected at revision, so please note any specific errors here.

Reviewer #1: Yes
________________________________

5. Review Comments to the Author

Please use the space provided to explain your answers to the questions above. You may also include additional comments for the author, including concerns about dual publication, research ethics, or publication ethics. (Please upload your review as an attachment if it exceeds 20,000 characters)

Reviewer #1: Review

OVERALL: I RECOMMEND THIS PAPER BE ACCEPTED WITH REVISIONS. SEE BELOW. I BELIEVE THIS RESEARCH IS RELEVANT, BUT THE AUTHORS WOULD BENEFIT FROM A MORE COMPLEX UNDERSTANDING OF THE PSYCHOLOGICAL AND RELATIONAL/ATTACHMENT MECHANISMS THAT CONTRIBUTE TO PTSD AND PTG.

**RESPONSE: We are grateful to the reviewer for their helpful comments and recommendation to publish subject to revisions. Our response to each comment made are below:**

5.1.

Abstract, and general comment: Why call it “deliberate rumination” rather than “deep, continuous thinking”, “reflective thinking”, or the like. Rumination has a negative connotation, at least for many psychotherapists. It tends to be associated with anxious thinking that is stuck, “rat on the wheel” thinking, that doesn’t get traction and make forward movement. The rumination that comes with PTSD is not the same as “deep, continuous thinking or problem solving”.

**RESPONSE: We acknowledge the reviewer comments in relation to the terminology used, however we would also highlight the inconsistent use of both ‘rumination’ and ‘continuous thinking’ in the literature. Rumination itself may refer to a number of types of recurrent and repetitive thinking, to include reflection and brooding [1], sense making and problem solving [2]. Negative rumination is closely associated with depression and may therefore have negative connotations as highlighted by the reviewer, however, rumination that is related to specific events are not necessarily always negative. Within the context of trauma, active ruminative thinking about an event is theorised to aid the cognitive processing of an event and ways to make sense of it, leading to growth [3-4], whereas some components of rumination about an event can be risk factors for distress. Due to this complexity, a distinction between intrusive and deliberate rumination was proposed [5] to aid understanding of the ruminative process and trauma. Intrusive rumination is not controlled by the individual, focuses on the negative aspects of experience, and can increase distress by thwarting the meaning-making process, whereas deliberate rumination may promote more positive aspects of events as it considers all aspects of the experience. Therefore, deliberate rumination goes beyond “deep continuous thinking” or “reflective thinking” as it is a cognitive process that involves deliberately re-examining an event, that may promote finding meaning in an experience, helping to develop understanding, value and significance from an event [6]. Given these considerations we have chosen to use the terminology ‘deliberate’ and ‘intrusive’ rumination in line with the literature related to post-traumatic growth. We therefore acknowledge the reviewer comments but have not changed the terminology in the manuscript.**

5.2

p. 2-3: I think the definition of trauma should include not only physical/psychological threat, but mind/body overwhelm, that is more than the person can bear given their consttitution, developmental status, intra-personal and inter-personal resources, etc. at the time of the event. If no overwhelm, no helplessness and powerlessness in the face of the event, then no trauma.

**RESPONSE: We agree and have expanded in line with the DSM-IV definition of trauma**

**Line 36:** “…and must elicit a response of intense fear, helplessness or terror”.

5.3

p. 4: While trauma’s impact on cognition and beliefs is very important, it is equally important to recognize the contribution of somatic experience and emotions on the survivor’s post-traumatic experience. I would at minimum reference the somatic and emotional domains of experience, in addition to the cognitive/beliefts.

**RESPONSE: We agree with the reviewer that a broader definition in relation to traumatic stress response should be considered and have included the following in the main body of the text**

**Line 41-43: “**The subjective severity of the event activates emotional distress, and often the distress this causes can prompt individuals to try to make sense of the meaning of the traumatic event(s) to relieve the distress [4, 5].”

5.4

p. 4: Again, intrusive thoughts are always accompanied by intrusive somatic experience and affect. It would important not to leave out the somatic/affective in this discussion, even though it is not the authors’ research focus.

**RESPONSE: We have included a clearer definition in line with the PTSD criterion to highlight the reviewer comment, as well as expanding on the definition of IR.**

**Line 45-49:** **“**PTSD may be defined by three clusters of symptoms: the re-experiencing of the traumatic event, persistent avoidance of trauma-related stimuli, and heightened persistent symptoms arousal. Memory, affect and response to trauma-stimuli are all effected, with the re-experiencing of the event (e.g., experiencing flashbacks) often with intense emotional experience (e.g., panic) considered a central component of PTSD[2]”.

**Line 113-117:** IR is a necessary diagnostic criterion for PTSD [29], to include intrusions of persistently re-experiencing via memories, nightmares, flashbacks, emotional distress or physical reactivity after exposure to traumatic reminders [2]. These intrusive thoughts focus on negative aspects and affect of the event and can then prompt the individual to process the trauma in a more conscious and voluntary cognitive manner via somatic cues;

5.5

p. 4-5: While I don’t like the term “deliberative rumination”, I think the key to its distinction from “intrusive rumination” is a) DR’s ability to change rather than remain repetitive and stuck; and 2) the DR person’s capacity for self-reflection. Without the capacity for self-reflection, that is to think about and give new meaning to traumatic experience, there can be no successful processing of the trauma, and therefore no post-traumatic group (ptg).

**RESPONSE: We have defined both IR and DR in line with current accepted definition within the PTG model and feel this is already addressed in the manuscript (definition of IR is expanded as per comment 5.5, DR definition unchanged from line 154).**

5.6

p. 5: While perhaps not absolutely necessary, I think it would be interesting if the authors’ briefly hypothesize as to why spirituality is associated with lower levels of distress, particularly post-trauma.

**RESPONSE: We have added a brief note to support what is already articulated about the research related to spirituality and PTG to address this.
line 177: “…**as spirituality can be seen as a source of support and guidance in the face of adversity”

5.7

p. 6: “1.Controlling for demographic variables, PTSD and IR, it is predicted that TST, DR and spirituality will all show unique positive associations with PTG.

What is “TST”? (Also, on p. 8, p. 14, 21, etc.)

**RESPONSE: TST is defined on page 4 as ‘time since trauma’ (line 124). Once defined, the acronym is used throughout. We have therefore not defined again on page 8, 14 etc.**

5.8

p. 7: “Inclusion criteria: University students were eligible to take part in the survey if they were over 18 years and had experienced a traumatic life event after the age of 16 years. This age limit was chosen in an attempt to capture incidents of Type I trauma (rather than complex Type II trauma), and to ensure that participants had cognitively processed the trauma as adults and not as children.”

I’d recommend the authors explain why they were focusing on Type 1 trauma.

**RESPONSE: We have expanded on our consideration of why Type I trauma is more associated with PTG in the introduction**

**Line 73-76: “**This may be due to the theoretical assumption that events need to be of significance to trigger cognitive rumination, processing, and emotion regulation [6], but not so significant (for example, repeated and prolonged trauma) as to hinder the process [17]”.

**And added clarity in the method**

**Line 202: “**(rather than complex Type II trauma which is less associated with PTG)”

Also, please specify what percentage were Type I vs Type II trauma.

**RESPONSE: We do not have this data, as only those with Type I trauma were included as eligible for the study. Those who met criteria for more complex trauma were excluded at screening. No changes are therefore made in light of this comment.**

I would also consider comparing interpersonal vs. non-human (e.g. car accident, natural disaster, illness) trauma, as that could potentially differentiate with respect to PTG and the effects of spirituality.

**RESPONSE: We thank the reviewer for this comment, and whilst we acknowledge this may be a useful exploration in the wider literature, we were primarily interested in the cognitive process regardless of the event categorisation in the current manuscript. Given this we only explored ‘potential’ trauma type and did not collect much data in relation to the categorisation of events. For those who did indicate type of trauma, the numbers are relatively small and unequal and therefore would limit the conclusions drawn from subsequent analyses. We therefore have not made any changes in light of this comment.**

5.9
p. 9: “The Event Related Rumination Inventory (ERRI; [26]) is a 20-item scale used to assess levels of rumination during the weeks immediately following a highly stressful event.”

Since this measure assesses the degree of rumination weeks after the stressful event, how do you know this is an accurate measure 6 months later, for example. The authors should explain how they understand the ERRI is still appropriate 6 months later.

**RESPONSE: At the time of conducting the study, there was only one questionnaire that measured intrusive and deliberate rumination response to a specific and traumatic event: the Event Related Rumination Inventory [11]. This measure is widely used in the literature. The questionnaire was used in line with measure guidelines, in exploring rumination in the weeks following an event. Although an element of recall could be unreliable, as we state in our discussion (see line 537), the internal consistency in the current study is in line with those reported elsewhere. We have added a statement to reflect this in the limitations section.**

**Line 538-539: “**Nevertheless, internal consistency of the measures in the current study are comparable to those reported in other studies”.

5.10

pp. 10-11: With respect to the spirituality measure, the authors should explain if they used the overall score, as contrasted with the subscales, and why, as pertains to their research questions.

**RESPONSE: Throughout, when referring to ‘spirituality’, the total score is used in line with the hypotheses posed. Where subscales are considered in terms of descriptive explorative data, this has been stated in the text. To clarify we have indicated that ‘total scores’ were used in the data analysis section for the moderator analyses (Lines 336 and 340).**

5.11

p. 17: “Additionally, a significant positive relationship was found between DR and spirituality. This suggests that greater the spirituality, the more likely they are to engage in DR, and conversely, the more someone engages in DR, the more likely they are to report having spiritual beliefs.”

 It is important the authors’ hypothesize why they believe there is a significant positive relationship between DR and spirituality. What is the cognitive, emotional, and/or relational, etc. explanation for the correlation.

**RESPONSE: We agree that this should be expanded upon. We have done so in the discussion section, rather than in the results section as suggested here to allow space for greater elaboration.**

**Line 505-518:** “The PTG model proposes that challenges to core beliefs is the causal component that triggers cognitive processing that leads to PTG [3]. Here, DR, actively reconstructing and rebuilding understandings of the self, others, and the world are under investigation and lead to positive post-trauma changes [56]. For those who hold spiritual beliefs as important, being able to use this as a framework from which to explore their core beliefs may be a pathway where an interplay of social, emotional and cognitive processes allow growth the occur. That is, that spirituality may help set DR in motion to process the event and facilitate growth as belief systems can provide frameworks to assimilate challenging life circumstances into one’s existing core beliefs [33]. Being supported by a social/spiritual community may lessen feelings of isolation or grief, forgiveness may be practiced more than revenge, physiological relaxation and reflection may be enhanced through prayer/meditation, and fewer risky behaviours (such as drinking/smoking as coping strategies) may encourage DR in processing the traumatic event”.

5.12
p. 17: “This finding suggests that spirituality is a moderator in the relationship between DR and PTG.”

It is important the authors’ hypothesize why they believe there is a significant positive relationship between DR and spirituality. What is the cognitive, emotional, and/or relational, etc. explanation for the correlation.

**RESPONSE: This is addressed as our response to 5.11**

5.13

p. 20: “However, the moderating relationship reported here supports the theory that having a belief system can provide individuals with a framework that facilitates DR and attempts at understanding and meaning-making in the aftermath of distressing life events [31].”

While this may be true, the authors’ should think about other ways DR and spirituality contribute to PTG. For example, might these positive correlations have something to do with a self-reflective capacity, seen in both DR and spirituality? Might spirituality allow a person to feel a connection with God/higher power, and this provides a secure attachment to helps the trauma survivor feel less alone, and therefore less likely to feel overwhelmed by trauma reminders. In short, I’d recommend the authors’ consider various psychological variables other than “meaning making” alone that might best explain these correlations. At minimum, I’d recommend the authors’ offer alternative explanations/hypotheses explaining the positive correlations, under the rubric of “Further Study” (pp. 21-22).

**RESPONSE: We have expanded as above, and included further comment in the further study section.**

**Line 577-588:** “Exploring such concepts more fully via qualitative accounts of spirituality post-trauma would be useful to provide perspectives to the proposed frameworks suggested here. For example, meaning-making may act as one explanation for the relationship between DR, spirituality and growth, however, other aspects of spirituality are worth exploring. Positive aspects of belonging to a spiritual community, a sense of mastery and control in accepting unalterable circumstances, or closeness and comfort in having a spiritual connection to a higher power may explain aspects of the experience”.

5.14

p. 20: “The subscale of ‘existential well-being’ appears to be an anomaly amongst the spirituality subsections.”

I’d recommend the authors look at the specific items that make up the “existential well-being” subscale. That might give them greater or less support for their hypothesis. For example, I could imagine “existential” might have more to do with the individual alone, than connected with a higher power. If that were the case, then “alone” vs. “securely attached with an higher power” could significantly impact PTSD and PTG.

**RESPONSE: As the reviewer suggests, the subscale is phrased more towards the individual – we have included this to further support our discussion here.**

**Line 471-473**

In considering the items that comprise the subscale, items are phrased with more of a focus on the individual alone. With that in mind, existential well-being may be akin to resilience

5.15

p. 20: “Consistent with existing literature, a strong positive relationship was found between DR and PTG.” There may be other explanations to explain this correlation than the one offered by the authors. For example, DR is associated with a capacity for self-reflection, and this capacity to “think about” the self and the traumatic experience could attenuate the PTSD symptoms and enhance PTG. At minimum, I’d like the authors to hypothesize about possible alternative explanations for the DR/PTG correlation, and include this again under “Further Study” (pp. 21-22).

**RESPONSE: We have included some further consideration about the relationship in the discussion.**

**Line 488-491: ‘**Engaging in DR is proposed as a protective factor against PTSD and a predictive factor for PTG [54]. This may be due to the fact that DR, as a deliberate re-examining of the self and the event, can reduce trauma related fear, attenuating the PTSD symptoms and enhancing PTG [54-55].

5.16

p. 21: “However, measures of reflective and brooding rumination mostly relate to depression rather than in relation to traumatic events.”
I’d recommend the authors reconsider the power of reflection vs brooding as relates to trauma. Reflective vs perseverative thinking is, in my clinical experience, differentially correlated with PTSD and PTG. In short, I would predict the greater reflective capacity, the less PTSD and greater PTG symptoms.

**RESPONSE: We agree this requires some broader reflection, and have included this in the discussion.**

**Line 544-565:** “DR and IR in the context of trauma refers specifically to the cognitive processing of an event and its consequences, whereas rumination in the context of depression is more associated with depressive symptoms/mood and its consequences, rather than life events [58-59]. Adopting measures that explore reflection and perseverative thinking, for example, may further elucidate the relationship of thinking styles in addition to cognitive processing, meaning making, and problem solving for those who have experienced traumatic events.

Theoretically, the findings may contribute to our understanding of the nature of both emotional and ruminative processes. Arguments exist over whether the subtypes within these processes (i.e. positive and negative emotions, and deliberate and intrusive rumination) exist at opposite ends of a spectrum or are distinct constructs. Vazquez [60] argues that positive and negative emotions are independent constructs which can co-exist, and Taku, Cann, Tedeschi & Calhoun [61] recommend viewing the subtypes of rumination as multi-dimensional, separate constructs, which may overlap but are essentially distinct. The findings of the current study lend support to the distinct constructs theories of both rumination and emotion, as different cognitive variables were found to contribute to both the PTG model (with its associated positive emotions) and PTSD model (with its associated negative emotions). However, Salsman, Segerstrom, Brechting, Carlson and Andrykowski [62 p. 39] state that "much work remains to further delineate the nature of cognitive processing” as the available empirical literature is inconclusive”.

5.17

p. 22: “Even if clients do not hold spiritual beliefs, therapy could be tailored to encourage particular thinking styles that are associated with spirituality, such as acceptance [56] to aid reflective rumination and foster psychological growth.”

While thinking styles does impact PTG vs PTSD, in my clinical experience and that of many other, emotional and somatic experience, that is subcortical experience, are more important. The authors should at minimum mention this and recommend it for future research.

**RESPONSE: We have added a section to consider further therapies in the clinical implications section.**

**Line 501-504:** “Additionally, therapies such as Eye-Movement Desensitization and Reprocessing (EMDR) can support the development of DR by facilitating a purposeful exploration of post-traumatic symptoms, supporting the exploration of the mechanisms of the traumatic response in terms of emotion regulation, threat, and memory [67]”.

**ADDITIONAL AMENDMENTS**

After revision of author guidelines and discussion with the authors, Dr Gundi Kiemle is no longer listed as an author but acknowledged for their contribution.

**REFERENCES (cited in response letter)**

1. Treynor W, Gonzalez R, Nolen-Hoeksema S. Rumination reconsidered: psychometric analysis. Cog Ther Res. 2003; 27:247–259
2. Martin LL, Tesser A. Some ruminative thoughts. In Wyer RS Jr, editor. Ruminative thoughts: Advances in Social Cognition (vol 9). Mahwah, NJ: Lawrence Erlbaum Associates. 1996. p 1–47.
3. Tedeschi RG, Calhoun L. Posttraumatic Growth: Future Directions. In: Tedeschi RG, Park CL Calhoun LG (editors). Post-Traumatic Growth: Positive Changes in the Aftermath of Crisis. Mahwah: Lawrence Erlbaum. 1998, p 93-102.
4. Calhoun, LG, Tedeschi, RG. The Foundations of Posttraumatic Growth: An Expanded Framework. In Calhoun LG, Tedeschi RG, editors. Handbook of Posttraumatic Growth, Research and Practice. Mahwah, NJ: Lawrence Erlbaum Associates. 2006. p 3-23.
5. Cann A, Calhoun LG, Tedeschi RG, Taku K, Vishnevsky T, Triplett KN, Danhauer SC. A short form of the posttraumatic growth inventory. Anxiety Stress Coping. 2010;23:127–137. doi: 10.1080/10615800903094273
6. Cann A, Calhoun LG, Tedeschi RG, Triplett KN, Vishnevsky T, Lindstrom CM Assessing posttraumatic cognitive processes: the event related rumination inventory. Anxiety Stress Coping. 2011; 24:137–156
7. Kamijo N, Yukawa S. The Role of Rumination and Negative Affect in Meaning Making Following Stressful Experiences in a Japanese Sample. Front Psychol. 2018;9:2404. https://doi.org/10.3389/fpsyg.2018.02404

6. PLOS authors have the option to publish the peer review history of their article (what does this mean?<<https://hes32-ctp.trendmicro.com:443/wis/clicktime/v1/query?url=https%3a%2f%2fjournals.plos.org%2fplosone%2fs%2feditorial%2dand%2dpeer%2dreview%2dprocess%23loc%2dpeer%2dreview%2dhistory&umid=3ce35460-db02-4393-9388-706b34028785&auth=768f192bba830b801fed4f40fb360f4d1374fa7c-44600eff4c732db3640dd20ed070ffec39a098d0>>). If published, this will include your full peer review and any attached files.

If you choose “no”, your identity will remain anonymous but your review may still be made public.

Do you want your identity to be public for this peer review? For information about this choice, including consent withdrawal, please see our Privacy Policy<<https://hes32-ctp.trendmicro.com:443/wis/clicktime/v1/query?url=https%3a%2f%2fwww.plos.org%2fprivacy%2dpolicy&umid=3ce35460-db02-4393-9388-706b34028785&auth=768f192bba830b801fed4f40fb360f4d1374fa7c-82b8a28593e17ad43fac8ad5b105702aa08c0a95>>.

Reviewer #1: No
[NOTE: If reviewer comments were submitted as an attachment file, they will be attached to this email and accessible via the submission site. Please log into your account, locate the manuscript record, and check for the action link "View Attachments". If this link does not appear, there are no attachment files.]

While revising your submission, please upload your figure files to the Preflight Analysis and Conversion Engine (PACE) digital diagnostic tool, <https://hes32-ctp.trendmicro.com:443/wis/clicktime/v1/query?url=https%3a%2f%2fpacev2.apexcovantage.com&umid=3ce35460-db02-4393-9388-706b34028785&auth=768f192bba830b801fed4f40fb360f4d1374fa7c-2a6215b1b14cbdc9aa27a4f955b6413c5f5d1a17>. PACE helps ensure that figures meet PLOS requirements. To use PACE, you must first register as a user. Registration is free. Then, login and navigate to the UPLOAD tab, where you will find detailed instructions on how to use the tool. If you encounter any issues or have any questions when using PACE, please email PLOS at [figures@plos.org](mailto:figures@plos.org)<mailto:[figures@plos.org](mailto:figures@plos.org)>. Please note that Supporting Information files do not need this step.
________________________________
 In compliance with data protection regulations, you may request that we remove your personal registration details at any time. (Remove my information/details)<<https://hes32-ctp.trendmicro.com:443/wis/clicktime/v1/query?url=https%3a%2f%2fwww.editorialmanager.com%2fpone%2flogin.asp%3fa%3dr&umid=3ce35460-db02-4393-9388-706b34028785&auth=768f192bba830b801fed4f40fb360f4d1374fa7c-ec58063f735a6c39d670c78704c496829887dd5b>>. Please contact the publication office if you have any questions.
