# Supplementary material for: The role of repetitive thinking and spirituality in the development of posttraumatic growth and symptoms of posttraumatic stress disorder
Source: PLoS One. 2022 Aug 4;17(8):e0272036. doi: 10.1371/journal.pone.0272036 (PMC9352075; doi:10.1371/journal.pone.0272036)
Supplement: Attachment — Submitted filename: Review of Revised Version- PLOS One (KB) (11-12-21).docx [file pone.0272036.s003.docx]

Review of Revised Version:

PLOS ONE

The role of repetitive thinking and spirituality in the development of posttraumatic growth and symptoms of posttraumatic stress disorder --Manuscript Draft-- Manuscript Number: PONE-D-21-05935

Page 1

“The Posttraumatic stress [change to Stress] Diagnostic Scale…

Page 2

“These findings indicate that, although spirituality…”. Suggest you change to, “These findings indicate that while spirituality…”

Page 3

You wrote:

“Often the distress this causes can prompt individuals to try to make sense of the meaning of the traumatic event(s) [4] and failure to find meaning has been found to be associated with poor psychological outcomes, including depression and post-traumatic stress disorder (PTSD) [5].”

Suggest rewrite, to aid in clarity:

“Often the distress this causes can prompt individuals to try to make sense of the meaning of the traumatic event(s) [4]. Failure to find meaning has been found to be associated with poor psychological outcomes, including depression and post-traumatic stress disorder (PTSD) [5].”

…

You wrote:

“In contrast, finding meaning in the aftermath of trauma has the potential to experience profound positive psychological transformation and higher levels of psychological functioning than prior to the event(s).”

Suggest rewrite, for better word usage:

“In contrast, when people find meaning in the aftermath of trauma, they have the potential to experience profound positive psychological transformation and higher levels of psychological functioning than prior to the event(s).”

…

You wrote:

“PTG has been associated more with incidents of Type I trauma, rather than Type II [11] occurring following a wide range of Type I traumatic events, e.g. natural disasters [12]road traffic accidents [13] illnesses [14], and interpersonal violence [15].”

Recommend rewrite for clarity:

“PTG has been associated more with incidents of Type I trauma than Type II [11]. PTG has been shown to occur following a wide range of Type I traumatic events, including natural disasters [12], road traffic accidents [13], illnesses [14], and interpersonal violence [15].”

…

Page 4

You wrote, “…participants from the USA reporting higher levels of PTG than other countries, possibly due to social desirability of responding to challenges with positivity [16].”

Maybe this has something to do with the social, emotional, and financial resource to the survivor. Could add to your remark, above.

…

You wrote:

“…however most agree that it takes time, sometimes many years, to develop [25].”

For clarity, I would change to:

“… however most agree that it takes time, sometimes many years, for PTG to develop [25].”

…

“…‘deliberate rumination’ (DR, [26]).”

I know you are not going to change this term, but I think it is an unfortunate one. “Rumination” is thinking usually association with thinking, like worry, that goes over and over the same thing and does not make forward progress. “Reflection” would be a much better term, and closer to what most needs to happen. It leads to positive change, growth, transformation, etc. For example, see this:

<https://www.google.com/search?q=reflection+and+rumination&rlz=1C5CHFA_enUS806US806&oq=reflection&aqs=chrome.3.69i57j35i39j0i67i433j0i67l2j0i67i433j0i20i263i433i512j0i67j46i131i433i512j0i67.5083j0j9&sourceid=chrome&ie=UTF-8>

“Self**-**reflection is a tool that helps us approach life with a growth mindset. ... Where self-reflection is purposefully processing (thinking about) our experiences with the intent of learning something, rumination is when we think over and over about something in the past or future with negative emotions directly linked.”

I would recommend you indicate that you are using the term others have used, but that the DR would be better termed, “deliberate *reflection*”.

Page 5

You wrote: “Belief systems can provide frameworks that ***assimilate*** challenging life circumstances into one’s existing core beliefs [31].”

I recommend you change the word “assimilate” to “accommodate”. See this:

<https://www.google.com/search?q=assimilation+versus+accommodation&rlz=1C5CHFA_enUS806US806&oq=assimiliate+versus+accom&aqs=chrome.1.69i57j0i22i30j0i10i22i30j0i390.13783j1j9&sourceid=chrome&ie=UTF-8>

Assimilation is the **process of using or transforming the environment** so that it can be placed in preexisting cognitive structures. Accomodation is the process of changing cognitive structures in order to accept something from the environment.

My view comes from Piaget’s discussion of assimilation and accommodation.

You wrote: “Studies report mixed results in terms of ***its*** benefits,…”

Please make more clear what “its” refers to.

…

You wrote: “MacDonald et al. [35] expand on this definition, to include: *"spirituality is a natural aspect of human functioning…”*

This would read more clear: “MacDonald et al. [35] expand on this definition: *"Spirituality is a natural aspect of human functioning…”*

GENERAL COMMENT: Do not italicize quotes without indicating those are yours, by writing at end of quote-- (my emphasis).

…

Use comma not semi-colon in this sentence, as follows:

“Spirituality has been found to be a moderator of distress, with higher levels of spirituality associated with lower levels of distress, and lower levels of spirituality associated with higher levels of distress [36].”

…

Recommend rewording this sentence as follows:

“This study ~~objectives~~ explores mechanisms that can promote adaptive outcomes following exposure to traumatic events to inform clinical practice. The interactive relationships of spirituality with the subtypes of repetitive thinking in relation to PTG and PTSD are **also** explored.”

…

Page 6

Recommend rewording as follows, for greater clarity:

“The current study ~~aims to test if~~ **evaluates whether** 1) high levels of DR and high levels of spirituality are associated with PTG, ~~and whether~~ 2) spirituality modifies this relationship; and 3) ~~as well as exploring~~ whether the relationship between IR and PTSD is moderated by spirituality.”

…

Before you go to your hypotheses, I think it is important to explain, at least briefly, why you think spirituality and DR would be associated with PTG. Also, your abbreviations are easily forgotten, so I recommend you write the full word with the abbreviation when you reintroduce them, as you do here:

*“Hypotheses Relevant to PTG:”*

For example, I didn’t know what “TST” refered to, in this sentence, and had to look it up because you mention it only once before, I believe:

“Controlling for demographic variables, PTSD and IR, it is predicted that TST, DR and spirituality will all show unique positive associations with PTG.”

You wrote: “This age limit was chosen in an attempt to capture incidents of Type I trauma (rather than complex Type II trauma), and to ensure that participants had cognitively processed the trauma as adults and not as children.”

Studying Type 1 versus Type 2 is a significant choice, as DR and spirituality may not have the same impact on PTG. I would definitely explain your reasoning for your selective criteria, some.

…

Page 9

You wrote: “Two additional questions were included to ascertain whether they experienced a head injury during the event, and if they received any therapy in relation to the trauma as potential confounders in considering cognitive response to trauma.”

These additional questions are very valuable. Please explain your rationale for their inclusion, i.e., why they are important vis a vis your hypotheses.

…

Likewise, you wrote: “Item 39 (“How long have you experienced the problems that you reported above?”) and part 4 (“Indicate if the problems have interfered with any of the following areas of your life”) of the PDS were not included.”

Please indicate why you excluded these questions, as they look like they might be relevant to your hypotheses.

…

Page 10

I think you need quotation marks for the test items, as I corrected below:

Items 31 (“This questionnaire appears to be measuring spirituality”) and 32 (“I responded to all statements honestly”) were not included in the current study, as they do not contribute to the calculation of final scores.

…

Page 13

You wrote: “The PDS indicates that the mean number of PTSD symptoms experienced was less than the normative PTSD patient sample, but the subscales were all more than a non-clinical sample.”

I haven’t read your discussion, yet, but it is important you make clear the PTSD symptoms you are assessing are not severe, i.e., comparable to most PTSD patients. Therefore, please stipulate that these findings do not necessarily apply to PTSD patients, which would need to be further evaluated.

…

Page 17

You wrote: “A moderate positive relationship was found between IR and symptoms of PTSD, suggesting that the more intrusive thoughts experienced, the more likely symptoms of PTSD are experienced.”

I’m not sure you want to include this, as the definition of PTSD includes intrusive thoughts.

…

You wrote: “A significant interaction (see Table 4) was found between the DR and spirituality (*b*=0.014, *SE_b_*=0.006, *t*=1.34, *p*=.02). This finding suggests that spirituality is a moderator in the relationship between DR and PTG.”

Why does this not suggest DR is the moderator between spirituality and PTG? This would help me to better understand your interpretation of this finding.

…

Page 18

You wrote: “…that the relationship between DR and PTG becomes stronger in people with average to high levels of spirituality (see Fig. 2 for the simple slopes graph).”

What is the correlation between DR and spirituality? Is there much overlap? I don’t recall if you assessed that. It would be important to do so, as you want to be sure your assessing distinct factors.

…

Page 19

You wrote: “This suggests that although spirituality is not associated with negative trauma outcomes, it is related to positive trauma outcomes and may play a role in facilitating growth following a traumatic experience. These findings contradict previous findings that higher levels of spirituality are associated with lower levels of distress and lower levels of spirituality are associated with higher levels of distress (e.g., [36]).”

Since your finding differs significantly from previous findings, i.e., positive vs negative effects of spirituality and PTG, it is important you provide a rationale for your distinct findings.

…

Page 20

You wrote: “Existential well-being may be akin to resilience (see [42]), which enables coping when faced with distress, but does not help an individual to grow following a trauma.”

This feels like a stretch to me. At minimum, you would have to explain what statements correspond with “existential well-being”, and what descriptors are used to describe “resilience”, so I better understand how these two factors might be correlated. As it is written here, I am not finding your suggestion compelling.

…

You wrote: “DR explained a significant amount of the PTG regression model and was also found to be the strongest predictor of PTG amongst the variables tested, further supporting Calhoun and Tedeschi’s [51] theoretical model of DR as an important cognitive process in the development of PTG.”

I think this is, perhaps, THE most important finding of your study. You need to explain the relationship, in your view, between DR and PTG. That is, what exactly is the cognitive process of DR that facilitates PTG. Very important, in my view.

…

You wrote: “As predicted, IR was positively associated with symptoms of PTSD, with IR accounting for almost a quarter of the variance in PTSD, supporting the evidence for these processes as involved in processing traumatic experience.”

As I noted above, this hypothesis states that a symptom of PTSD, IR, is a symptom of PTSD. I do not see the value of this hypothesis and finding.

…

Page 21

You wrote: “No association was found between PTG and gender, contradicting the existing literature (e.g. [21]) that suggests females are more likely to experience PTG.”

Again, since this finding is different than previous findings, it would be helpful for you to briefly explain why this is so.

…

Page 22

Typo, line 472. Should be capitalized: “However, “

…

Under **Limitations** of this study, I think it is very important that you specify that you are studying Type 1 (simple trauma) and not Type 2 (complex trauma). I believe type 2 trauma is what most trauma therapists see and treat, more often.

…

You wrote: “As Taylor[54] argues, psychology needs to find a scientific method of measuring value systems or it risks losing the human element from which it stemmed, especially in regards to the subject of trauma, when values and beliefs of this kind are often relied on for comfort and strength.”

This is a very important statement. Well done.

…

I think several of your clinical implications are of value. However, you wrote:

“Belief systems can be used as a framework and context in which to promote DR in order to derive some benefit from distress [55].”

You would need to flesh this out much more if you are to make this statement. Please explain how “belief systems” “promote DR”. At least offer one way this may be so, and indicate this can be further researched.
