# Supplementary material for: The role of repetitive thinking and spirituality in the development of posttraumatic growth and symptoms of posttraumatic stress disorder
Source: PLoS One. 2022 Aug 4;17(8):e0272036. doi: 10.1371/journal.pone.0272036 (PMC9352075; doi:10.1371/journal.pone.0272036)
Supplement: Attachment — Submitted filename: Response to reviewers.docx [file pone.0272036.s004.docx]

Dear Editor

Thank you for the opportunity to further respond to the reviewer comments in relation to our article “The role of repetitive thinking and spirituality in the development of posttraumatic growth and symptoms of posttraumatic stress disorder”. Please see below each comment numbered, with indicative line numbers for amendments noted underneath in bold. All amendments are tracked in the manuscript.

Please note for some text quoted by the reviewer, incorrect citation numbers are given. These have not been highlighted as an amendment as they do not differ in the previous and current version of the manuscript.

We would also like to extend our thanks to the reviewer for a detailed and informative review, which has strengthened our manuscript.

We look forward to hearing from you.

Best wishes

Dr Catrin Eames, on behalf of the authors.

**Reviewer comments**

1. Page 1

“The Posttraumatic stress [change to Stress] Diagnostic Scale…

**RESPONSE: Amended, line 8.**

2. Page 2

“These findings indicate that, although spirituality…”. Suggest you change to, “These findings indicate that while spirituality…”

**RESPONSE: Amended, line 14.**

3. Page 3

You wrote:

“Often the distress this causes can prompt individuals to try to make sense of the meaning of the traumatic event(s) [4] and failure to find meaning has been found to be associated with poor psychological outcomes, including depression and post-traumatic stress disorder (PTSD) [5].”

Suggest rewrite, to aid in clarity:

“Often the distress this causes can prompt individuals to try to make sense of the meaning of the traumatic event(s) [4]. Failure to find meaning has been found to be associated with poor psychological outcomes, including depression and post-traumatic stress disorder (PTSD) [5].”

**RESPONSE: Amended, lines 41-43 (note phrasing in the manuscript differed somewhat to what the reviewer notes here). Amended in line with reviewer suggestion.**

4. You wrote:

“In contrast, finding meaning in the aftermath of trauma has the potential to experience profound positive psychological transformation and higher levels of psychological functioning than prior to the event(s).”

Suggest rewrite, for better word usage:

“In contrast, when people find meaning in the aftermath of trauma, they have the potential to experience profound positive psychological transformation and higher levels of psychological functioning than prior to the event(s).”

**RESPONSE: Amended, line 53.**

5. You wrote:

“PTG has been associated more with incidents of Type I trauma, rather than Type II [11] occurring following a wide range of Type I traumatic events, e.g. natural disasters [12]road traffic accidents [13] illnesses [14], and interpersonal violence [15].”

Recommend rewrite for clarity:

“PTG has been associated more with incidents of Type I trauma than Type II [11]. PTG has been shown to occur following a wide range of Type I traumatic events, including natural disasters [12], road traffic accidents [13], illnesses [14], and interpersonal violence [15].”

**RESPONSE: Amended lines 70-71 (note citation numbers differ in reviewer comments to that noted in the manuscript).**

6. Page 4

You wrote, “…participants from the USA reporting higher levels of PTG than other countries, possibly due to social desirability of responding to challenges with positivity [16].”

Maybe this has something to do with the social, emotional, and financial resource to the survivor. Could add to your remark, above.

**RESPONSE: Amended, lines 78-83.**

**For example, the social pressure to report growth from adversity is greater in trauma survivors from the USA than those in Europe, and may therefore be a cultural expectation [18]. This is noted regardless of social factors such as non-Caucasian ethnicity and lack of education, both of which are associated with higher growth and are risk factors for PTSD, whereas lack of social support is associated with less growth [16].**

7. You wrote:

“…however most agree that it takes time, sometimes many years, to develop [25].”

For clarity, I would change to:

“… however most agree that it takes time, sometimes many years, for PTG to develop [25].”

**RESPONSE: Amended, line 95.**

8. “…‘deliberate rumination’ (DR, [26]).”

I know you are not going to change this term, but I think it is an unfortunate one. “Rumination” is thinking usually association with thinking, like worry, that goes over and over the same thing and does not make forward progress. “Reflection” would be a much better term, and closer to what most needs to happen. It leads to positive change, growth, transformation, etc. For example, see this:

<https://www.google.com/search?q=reflection+and+rumination&rlz=1C5CHFA_enUS806US806&oq=reflection&aqs=chrome.3.69i57j35i39j0i67i433j0i67l2j0i67i433j0i20i263i433i512j0i67j46i131i433i512j0i67.5083j0j9&sourceid=chrome&ie=UTF-8>

“Self**-**reflection is a tool that helps us approach life with a growth mindset. ... Where self-reflection is purposefully processing (thinking about) our experiences with the intent of learning something, rumination is when we think over and over about something in the past or future with negative emotions directly linked.”

I would recommend you indicate that you are using the term others have used, but that the DR would be better termed, “deliberate *reflection*”.

**RESPONSE: We have included the following to reflect the reviewer comments here. Lines 116-121.**

**In line with the literature related to PTG, we adopt the terminology of deliberate and intrusive rumination throughout the manuscript. Here, deliberate rumination is defined as a cognitive process that involves deliberately re-examining an event, that may promote finding meaning in an experience, helping to develop understanding, value and significance from an event. We acknowledge that deliberate reflection, the process of purposefully thinking about an experience with an intent to learn, can also be applied within this context.**

9. Page 5

You wrote: “Belief systems can provide frameworks that ***assimilate*** challenging life circumstances into one’s existing core beliefs [31].”

I recommend you change the word “assimilate” to “accommodate”. See this:

<https://www.google.com/search?q=assimilation+versus+accommodation&rlz=1C5CHFA_enUS806US806&oq=assimiliate+versus+accom&aqs=chrome.1.69i57j0i22i30j0i10i22i30j0i390.13783j1j9&sourceid=chrome&ie=UTF-8>

Assimilation is the **process of using or transforming the environment** so that it can be placed in preexisting cognitive structures. Accomodation is the process of changing cognitive structures in order to accept something from the environment.

My view comes from Piaget’s discussion of assimilation and accommodation.

**RESPONSE: Amended, line 127.**

10. You wrote: “Studies report mixed results in terms of ***its*** benefits,…”

Please make more clear what “its” refers to.

**RESPONSE: Amended line 128– now reads “Studies report mixed results, with belief systems associated with both positive and negative health outcomes”**

11. You wrote: “MacDonald et al. [35] expand on this definition, to include: *"spirituality is a natural aspect of human functioning…”*

This would read more clear: “MacDonald et al. [35] expand on this definition: *"Spirituality is a natural aspect of human functioning…”*

**RESPONSE: Amended, 133.**

12. GENERAL COMMENT: Do not italicize quotes without indicating those are yours, by writing at end of quote-- (my emphasis).

**RESPONSE: Amended – quotes now not italicized, lines 132, 133-138.**

13. Use comma not semi-colon in this sentence, as follows:

“Spirituality has been found to be a moderator of distress, with higher levels of spirituality associated with lower levels of distress, and lower levels of spirituality associated with higher levels of distress [36].”

**RESPONSE: Amended, line 139.**

14. Recommend rewording this sentence as follows:

“This study ~~objectives~~ explores mechanisms that can promote adaptive outcomes following exposure to traumatic events to inform clinical practice. The interactive relationships of spirituality with the subtypes of repetitive thinking in relation to PTG and PTSD are **also** explored.”

**RESPONSE: Amended, lines 149-151.**

15. Page 6

Recommend rewording as follows, for greater clarity:

“The current study ~~aims to test if~~ **evaluates whether** 1) high levels of DR and high levels of spirituality are associated with PTG, ~~and whether~~ 2) spirituality modifies this relationship; and 3) ~~as well as exploring~~ whether the relationship between IR and PTSD is moderated by spirituality.”

**RESPONSE: Amended, lines 152-153.**

16. Before you go to your hypotheses, I think it is important to explain, at least briefly, why you think spirituality and DR would be associated with PTG.

**RESPONSE: Amended, lines 154-156.**

**For those who hold spiritual beliefs as important, spirituality may provide a framework from which growth may occur via an interplay of cognitive, emotional, and social processes to explore core beliefs and challenging life circumstances.**

17. Also, your abbreviations are easily forgotten, so I recommend you write the full word with the abbreviation when you reintroduce them, as you do here:

*“Hypotheses Relevant to PTG:”*

For example, I didn’t know what “TST” refered to, in this sentence, and had to look it up because you mention it only once before, I believe:

“Controlling for demographic variables, PTSD and IR, it is predicted that TST, DR and spirituality will all show unique positive associations with PTG.”

**RESPONSE: Amended, lines 160-163.**

18. You wrote: “This age limit was chosen in an attempt to capture incidents of Type I trauma (rather than complex Type II trauma), and to ensure that participants had cognitively processed the trauma as adults and not as children.”

Studying Type 1 versus Type 2 is a significant choice, as DR and spirituality may not have the same impact on PTG. I would definitely explain your reasoning for your selective criteria, some.

**RESPONSE: This was already expanded upon in the previous revision, stating that Type II trauma is less associated with PTG – see highlight lines 193-194. No further amendment made.**

19. Page 9

You wrote: “Two additional questions were included to ascertain whether they experienced a head injury during the event, and if they received any therapy in relation to the trauma as potential confounders in considering cognitive response to trauma.”

These additional questions are very valuable. Please explain your rationale for their inclusion, i.e., why they are important vis a vis your hypotheses.

**RESPONSE: Amended to include the following text, lines 226-228.**

Two additional questions were included to ascertain whether they experienced a head injury during the event, and if they received any therapy in relation to the trauma. **These were included** as **both factors were deemed important to include as** potential confounders **when measuring** cognitive response to trauma.

…

20. Likewise, you wrote: “Item 39 (“How long have you experienced the problems that you reported above?”) and part 4 (“Indicate if the problems have interfered with any of the following areas of your life”) of the PDS were not included.”

Please indicate why you excluded these questions, as they look like they might be relevant to your hypotheses.

**RESPONSE: Amendment, lines 235-283.**

**Due to experimental error**, item 39 (“How long have you experienced the problems that you reported above?”) and part 4 (“Indicate if the problems have interfered with any of the following areas of your life”) of the PDS were **missing from the final online survey and therefore** were not included.

21. Page 10

I think you need quotation marks for the test items, as I corrected below:

Items 31 (“This questionnaire appears to be measuring spirituality”) and 32 (“I responded to all statements honestly”) were not included in the current study, as they do not contribute to the calculation of final scores.

**RESPONSE: Amended, line 264-265.**

22. Page 13

You wrote: “The PDS indicates that the mean number of PTSD symptoms experienced was less than the normative PTSD patient sample, but the subscales were all more than a non-clinical sample.”

I haven’t read your discussion, yet, but it is important you make clear the PTSD symptoms you are assessing are not severe, i.e., comparable to most PTSD patients. Therefore, please stipulate that these findings do not necessarily apply to PTSD patients, which would need to be further evaluated.

**RESPONSE: Amended, line 332:**

**These findings are therefore not representative of a patient sample.**

23. Page 17

You wrote: “A moderate positive relationship was found between IR and symptoms of PTSD, suggesting that the more intrusive thoughts experienced, the more likely symptoms of PTSD are experienced.”

I’m not sure you want to include this, as the definition of PTSD includes intrusive thoughts.

**RESPONSE: Amendment : removed in line with reviewer suggestion.**

24. You wrote: “A significant interaction (see Table 4) was found between the DR and spirituality (*b*=0.014, *SE_b_*=0.006, *t*=1.34, *p*=.02). This finding suggests that spirituality is a moderator in the relationship between DR and PTG.”

Why does this not suggest DR is the moderator between spirituality and PTG? This would help me to better understand your interpretation of this finding.

**RESPONSE: No amendment made as we feel we have introduced the analysis throughout the manuscript. To clarify further, the moderation analysis was conducted to test the hypothesis that spirituality would moderate the relationship between DR and PTG. Thus, spirituality was entered as a moderator variable. The simple slopes analysis indicates how the relationship between DR and PTG changes at different levels of spirituality (see Fig. 2). These results reveal that the relationship between DR and PTG becomes stronger as levels of spirituality increase, however, even when DR is low, levels of PTG are still higher in those with higher levels of spirituality compared to those with lower levels (see Fig. 2).**

25. Page 18

You wrote: “…that the relationship between DR and PTG becomes stronger in people with average to high levels of spirituality (see Fig. 2 for the simple slopes graph).”

What is the correlation between DR and spirituality? Is there much overlap? I don’t recall if you assessed that. It would be important to do so, as you want to be sure your assessing distinct factors.

**RESPONSE: This is reported in Table 1 as .36, which indicate a small to moderate relationship. No amendment made.**

26. Page 19

You wrote: “This suggests that although spirituality is not associated with negative trauma outcomes, it is related to positive trauma outcomes and may play a role in facilitating growth following a traumatic experience. These findings contradict previous findings that higher levels of spirituality are associated with lower levels of distress and lower levels of spirituality are associated with higher levels of distress (e.g., [36]).”

Since your finding differs significantly from previous findings, i.e., positive vs negative effects of spirituality and PTG, it is important you provide a rationale for your distinct findings.

**RESPONSE: Amendment: line 443-445** These findings contradict previous findings that higher levels of spirituality are associated with lower levels of distress and lower levels of spirituality are associated with higher levels of distress (e.g., [38]). **T**he moderating relationship reported here **provides a more nuanced understanding of this relationship** and supports the theory that having a belief system can provide individuals with a framework that facilitates DR and attempts at understanding and meaning-making in the aftermath of distressing life events [33].

27. Page 20

You wrote: “Existential well-being may be akin to resilience (see [42]), which enables coping when faced with distress, but does not help an individual to grow following a trauma.”

This feels like a stretch to me. At minimum, you would have to explain what statements correspond with “existential well-being”, and what descriptors are used to describe “resilience”, so I better understand how these two factors might be correlated. As it is written here, I am not finding your suggestion compelling.

**RESPONSE: Amendment lines 455-473.** In considering the items that comprise the subscale, items are phrased (**reverse scored**) with more of a focus on the individual alone **rather than a broader sense of spirituality (“It always seems that I am doing things wrong”, “I am not comfortable with myself”, “Much of what I do in life seems strained”, “My life is often troublesome”, “I often feel tense”, and “I am an unhappy person”**. Existential well-being **may be defined as subjective wellbeing in relation to meaning, purpose and satisfaction in life, as well as comfort in relation to death and suffering. When considering the items listed within this subscale, those reporting greater existential wellbeing report more acceptance and satisfaction with life. This may provide a platform where there is less need to make sense of events that are traumatic. We could consider existential wellbeing to function in a similar way as the construct of** resilience (see [45]), which enables coping when faced with distress, but does not necessarily help an individual to grow following a trauma. Resilience is inversely related to both PTSD and PTG [51]. Resilience has been defined as an individual’s ability to maintain equilibrium when experiencing aversive life circumstances [52], whereas PTG is an individual’s capacity to use the process of distress to enable them to make improvements in their life following a trauma [43]. Bonanno, Wortman and Nesse [53] propose that resilience provides a stability that results in less struggle with the aftermath of trauma, resulting in less of a need to make sense of events. **I**t may be possible that ‘existential well-being’ provides a similar function **which requires further exploration in the context of trauma.**

28. You wrote: “DR explained a significant amount of the PTG regression model and was also found to be the strongest predictor of PTG amongst the variables tested, further supporting Calhoun and Tedeschi’s [51] theoretical model of DR as an important cognitive process in the development of PTG.”

I think this is, perhaps, THE most important finding of your study. You need to explain the relationship, in your view, between DR and PTG. That is, what exactly is the cognitive process of DR that facilitates PTG. Very important, in my view

**RESPONSE: Amended line 489-491.**

**The deliberate re-examining and repetitive thinking about beliefs before and after trauma actively rebuilds understanding of an individuals’ place in, and assumptions of, a posttraumatic world.**

29. You wrote: “As predicted, IR was positively associated with symptoms of PTSD, with IR accounting for almost a quarter of the variance in PTSD, supporting the evidence for these processes as involved in processing traumatic experience.”

As I noted above, this hypothesis states that a symptom of PTSD, IR, is a symptom of PTSD. I do not see the value of this hypothesis and finding.

**RESPONSE: Amended - removed**

30. Page 21

You wrote: “No association was found between PTG and gender, contradicting the existing literature (e.g. [21]) that suggests females are more likely to experience PTG.”

Again, since this finding is different than previous findings, it would be helpful for you to briefly explain why this is so.

**RESPONSE: Amended lines 520-523**

**Such gender differences are more apparent with age, in that these differences increase as participants are older [23]. Given our sample were mostly young adults within a small age range, our distribution may be too narrow for such an effect.**

31. Page 22

Typo, line 472. Should be capitalized: “However, “

**RESPONSE: Amended line 527**

32. Under **Limitations** of this study, I think it is very important that you specify that you are studying Type 1 (simple trauma) and not Type 2 (complex trauma). I believe type 2 trauma is what most trauma therapists see and treat, more often.

**RESPONSE: Amended line 532-533, 539**

33. You wrote: “As Taylor[54] argues, psychology needs to find a scientific method of measuring value systems or it risks losing the human element from which it stemmed, especially in regards to the subject of trauma, when values and beliefs of this kind are often relied on for comfort and strength.”

This is a very important statement. Well done.

**RESPONSE: Thank you. No amendment made.**

34. I think several of your clinical implications are of value. However, you wrote:

“Belief systems can be used as a framework and context in which to promote DR in order to derive some benefit from distress [55].”

You would need to flesh this out much more if you are to make this statement. Please explain how “belief systems” “promote DR”. At least offer one way this may be so, and indicate this can be further researched.

**RESPONSE: Amended line 591-594.**

**For example, a starting point from which to make sense of the world, find meaning in experience, find the positives in distressing experiences and grow and develop psychologically in the aftermath of trauma.**

- **END OF DOCUMENT** -
