# Supplementary material for: The role of repetitive thinking and spirituality in the development of posttraumatic growth and symptoms of posttraumatic stress disorder
Source: PLoS One. 2022 Aug 4;17(8):e0272036. doi: 10.1371/journal.pone.0272036 (PMC9352075; doi:10.1371/journal.pone.0272036)
Supplement: Attachment — Submitted filename: Review of 2nd Revision (Repetitive Thinking) (KB, 2-12-22).docx [file pone.0272036.s005.docx]

Review of 2^nd^ Revision (Repetitive Thinking) (KB, 2-12-22)

PLOS ONE

The role of repetitive thinking and spirituality in the development of posttraumatic growth and symptoms of posttraumatic stress disorder

PONE-D-21-05935R2

Page 10, lines 208-210.

You wrote:

These were included as both factors were deemed important to include as potential confounders when measuring cognitive response to trauma.

Change wording to:

These were included as both factors were deemed important as potential confounders when measuring cognitive response to trauma.

Page 22, lines 428-432

Add parenthesis at end of sentence, as in:

In considering the items that comprise the subscale, items are phrased (reverse scored) with more of a focus on the individual alone rather than a broader sense of spirituality (“It always seems that I am doing things wrong”, “I am not comfortable with myself”, “Much of what I do in life seems strained”, “My life is often troublesome”, “I often feel tense”, and “I am an unhappy person”.)

Page 27, lines 547-548

Change wording, to make clearer the connection with the previous sentence, as in:

For example, belief systems can serve as a starting point from which to make sense of the world, find meaning in experience, find the…
