# Supplementary material for: The role of repetitive thinking and spirituality in the development of posttraumatic growth and symptoms of posttraumatic stress disorder
Source: PLoS One. 2022 Aug 4;17(8):e0272036. doi: 10.1371/journal.pone.0272036 (PMC9352075; doi:10.1371/journal.pone.0272036)
Supplement: Attachment — Submitted filename: Response to reviewers.docx [file pone.0272036.s006.docx]

Dear Editor

Thank you for the opportunity to further respond to the reviewer comments in relation to our article “The role of repetitive thinking and spirituality in the development of posttraumatic growth and symptoms of posttraumatic stress disorder” (PONE-D-21-05935R2). Please see below each comment numbered, with indicative line numbers for amendments noted underneath in bold. All amendments are tracked in the marked-up manuscript.

We have also checked the reference list in line with the journal requirements and have updated all doi formatting (**pages 29-32).**

We thank the reviewer for their detailed comments and are delighted to provide a revised version. We look forward to hearing from you with regard the revised version.

Best wishes

Dr Catrin Eames, on behalf of the authors.

**Reviewer comments**

1. Page 10, lines 208-210.

You wrote:

These were included as both factors were deemed important to include as potential confounders when measuring cognitive response to trauma.

Change wording to:

These were included as both factors were deemed important as potential confounders when measuring cognitive response to trauma.

**Response: Wording changed as advised (deleted ‘to include’), page 10, line 209.**

2. Page 22, lines 428-432

Add parenthesis at end of sentence, as in:

In considering the items that comprise the subscale, items are phrased (reverse scored) with more of a focus on the individual alone rather than a broader sense of spirituality (“It always seems that I am doing things wrong”, “I am not comfortable with myself”, “Much of what I do in life seems strained”, “My life is often troublesome”, “I often feel tense”, and “I am an unhappy person”.)

**Response: Parenthesis included, page 22, line 433.**

3. Page 27, lines 547-548

Change wording, to make clearer the connection with the previous sentence, as in:

For example, belief systems can serve as a starting point from which to make sense of the world, find meaning in experience, find the…

**Response: Wording changed as suggested (include ‘belief systems can serve as…’), page 27, line 548-9.**

**-END OF DOCUMENT-**
